# Community Ecology of Euglossine Bees in the Coastal Atlantic Forest of São Paulo State, Brazil

**DOI:** 10.1673/031.013.2301

**Published:** 2013-04-03

**Authors:** Léo Correia da Rocha-Filho, Carlos Alberto Garofalo

**Affiliations:** Departamento de Biologia, Faculdade de Filosofia, Ciências e Letras de Ribeirão Preto-FFCLRP, Universidade de São Paulo-USP, Av. Bandeirantes 3900, CEP 14040-901, Ribeirão Preto, SP, Brazil

**Keywords:** bioindicators, diversity, *Euglossa cordata*, orchid bees

## Abstract

The Atlantic Forest stretches along Brazil's Atlantic coast, from Rio Grande do Norte State in the north to Rio Grande do Sul State in the south, and inland as far as Paraguay and the Misiones Province of Argentina. This biome is one of the eight biodiversity hotspots in the world and is characterized by high species diversity. Euglossini bees are known as important pollinators in this biome, where their diversity is high. Due to the high impact of human activities in the Atlantic Forest, in the present study the community structure of Euglossini was assessed in a coastal lowland area, Parque Estadual da Serra do Mar - Núcleo Picinguaba (PESM), and in an island, Parque Estadual da Ilha Anchieta (PEIA), Ubatuba, São Paulo State, Brazil. Sampling was carried out monthly, from August 2007 to July 2009, using artificial baits with 14 aromatic compounds to attract males. Twenty-three species were recorded. On PEIA, *Euglossa cordata* (L.) (Hymenoptera: Apidae) represented almost two thirds of the total species collected (63.2%). *Euglossa iopoecila* (23.0%) was the most abundant species in PESM but was not recorded on the island, and *Euglossa sapphirina* (21.0%) was the second most frequent species in PESM but was represented by only nine individuals on PEIA. The results suggest that these two species may act as bioindicators of preserved environments, as suggested for other Euglossini species. Some authors showed that *Eg. cordata* is favored by disturbed environments, which could explain its high abundance on Anchieta Island. Similarly, as emphasized by other authors, the dominance of *Eg. cordata* on the island would be another factor indicative of environmental disturbance.

## Introduction

Euglossini bees (Hymenoptera: Apidae) are restricted to North, Central, and South America, with a distribution that extends from the southern United States and northern Mexico to the province of Córdoba, Argentina (Pearson and Dressier 1985; Minckley and Reyes 1996). Species of this tribe are common and are most diverse in the moist tropical and subtropical forests of Central and South America (Dressier 1982a). The tribe contains three pollen-collecting genera, *Euglossa* Latreille, *Eulaema* Lepeletier, and *Eufriesea* Cockerell, and two cleptoparasites genera, *Aglae* Lepeletier and Serville and *Exaerete* Hoffmannsegg, whose species have been reared from *Eulaema* spp. and *Eufriesea* spp. nests (Myers 1935; Myers and Loveless 1976; Kimsey 1987; Garófalo and Rozen 2001).

In Brazil, faunistic surveys of euglossine bees have been conducted in the Amazon region in the states of Amazonas (Becker et al. 1991; Morato et al. 1992; Oliveira and Campos 1995), Acre (Nemésio and Morato 2004), Roraima (Nemésio 2005), Mato Grosso (Anjos-da-Silva 2006), and Maranhão (Rebêlo and Cabrai 1997; Rebêlo and Silva 1999; Silva and Rebêlo 2002). Surveys have also been conducted in several Atlantic Forest remnants in the northeastern (Neves and Viana 1997, 1999; Bezerra and Martins 2001; Milet-Pinheiro and Schlindwein 2005), southeastern (Rebêlo and Garófalo 1991, 1997; Mateus et al. 1993; Wilms 1995; Garófalo et al. 1998; Bonilla-Gómez 1999; Peruquetti et al. 1999; Braga and Garófalo 2000; Camillo et al. 2000; Jesus and Garófalo 2000, 2004; Nascimento et al. 2000; Tonhasca et al. 2002; Knoll et al. 2004; Singer and Sazima 2004; Uehara-Prado and Prado and Garófalo 2006; Garófalo and Serrano 2008) and southern (Wittmann et al. 1988; Sofia et al. 2004; Sofia and Suzuki, 2004) regions of Brazil. However, the composition of the euglossine community remains poorly documented in several areas, including the coastal plains and slopes of the Serra do Mar of São Paulo State, which lies within the Atlantic Forest biome.

The Atlantic Forest stretches along Brazil's Atlantic coast, from Rio Grande do Norte State in the north to Rio Grande do Sul State in the south, and inland as far as Paraguay and the Misiones Province of Argentina. Once covering more than a million square kilometers, the forest has now been reduced to less than 8% of the original cover (SOS Mata Atlântica 1998). It is considered by the Conservation International as one of the world's biodiversity hot spots (Myers et al. 2000). The Atlantic Forest is composed of two major vegetation types, the coastal forest of Atlantic Rain Forest and the Tropical Semi-deciduous Forest. The Atlantic Rain Forest covers mostly the low to medium elevations (</= 1000 m.a.s.l.) of the eastern slopes of the mountain chain that runs along the coastline from southern to northeastern Brazil. The Atlantic Semi-deciduous Forest extends across the plateau (usually > 600 m.a.s.l.) in the center and southeastern interior of the country (Morellato and Haddad 2000).

In São Paulo State, a large portion of the largest and most significant area of the Atlantic Forest in Brazil is preserved and protected by the State Department of the Environment (Leitão-Filho 1994). This protected area includes “Parque Estadual da Serra do Mar”, which is part of the largest continuous portion of the Atlantic Forest in Brazil. The 315,000-hectare state park extends from the municipality of Itariri in the south to the state of Rio de Janeiro in the north.

Despite being a priority area for conservation, the Atlantic Forest biome remains severely threatened due to its proximity to urban centers and areas of agricultural monoculture such as coffee, orange, sugar cane, and eucalyptus plantations (Dean 1995; Jorge and Garcia 1997; Ranta et al. 1998; Morellato and Haddad 2000). In addition, the Atlantic Forest Hymenoptera fauna still remain virtually unknown, being less studied than that of the Brazilian open areas (Gonçalves and Brandão 2008).

The main purpose of our study was to examine the species richness, diversity, and abundance of male euglossine bees from two areas of the Atlantic Forest, Picinguaba and Anchieta Island, and characterized by different degrees of environmental preservation and human influence. Besides this information, we compared our data with those found in other areas of the Atlantic Forest in São Paulo State.

## Methods and Materials

### Study Areas

**Parque Estadual da Serra do Mar (PESM) — Nucleo Picinguaba.** The PESM covers an area of approximately 47,500 hectares in the municipality of Ubatuba. The park is administered by an operational center (“núcleo”) located in the district of Picinguaba, bordering the state of Rio de Janeiro. Núcleo Picinguaba contains the only section of the state park that reaches sea level and thus protects the local coastal ecosystems. This center is also surrounded by habitats representing nearly all of the Atlantic Forest ecosystems, from mangroves and coastal plain vegetation at the lower elevations to high-altitude grasslands at the highest points, which include the Pedra do Espelho (1,670 m.a.s.l.), Corcovado (1,150 m.a.s.l.), and Cuscuzeiro (1,275 m.a.s.l.) peaks in Ubatuba. Núcleo Picinguaba is situated in an environmentally strategic location at the boundary between PESM and Parque Estadual da Serra da Bocaina in Rio de Janeiro State. The Picinguaba district contains approximately 8,000 hectares of Atlantic Forest and is located in one of the most important tourist regions of the state of São Paulo, approximately 40 km from the municipality of Ubatuba.

**Parque Estadual da Ilha Anchieta (PEIA)**. Anchieta Island is located on the northern coast of São Paulo State (45′ 02° – 45′05° W and 23′31° – 23′ 34° S), approximately 600 m from the mainland just south of Ubatuba. The main access to the PEIA is via Palmas Bay, 8 km from the Saco da Ribeira marina in Flamengo Bay. The park occupies the entire 828-hectare island and has only one perennial stream, which is located in an area of coastal forest (Restinga). The topography is rugged and mountainous, with slopes typically greater than 24°. More level areas (with slopes under 6°) are found at two beaches (“Grande” and “Presídio”), and areas of intermediate slope are located in valley bottoms and on flatter hilltops on the island. The vegetation found on Anchieta Island has been described by Guillaumon et al. (1989) following Rizzini (1977) as including anthropic fields, rocky coast, Atlantic forest, Gleichenial, mangrove, and restinga (Peralta 2005).

### Methods

In both areas, the samples were made once a month from August 2007 to July 2009, between 09:00 and 15:00. Sampling was carried out along 50-m trails located on the edge of forested areas in succeeding days, i.e., one day in Picinguaba and the next day on Anchieta Island.

In the first year of study (August 2007 to July 2008), in Picinguaba, bees were collected along the Picadão da Barra trail (23° 21′ 51.7″ S and 44° 49′ 56.9″ W, 3 m.a.s.l.), which is located close to a state highway (BR 101). In the second year (August 2008 to July 2009), collection was conducted along the Guanambi trail (23° 21′ 37.0″ S and 44° 50′ 52.9″ W, 3 m.a.s.l.), located near the same highway (BR 101) at the main center of the Núcleo Picinguaba at Praia da Fazenda. The two trails used are separated by approximately 1.6 km.

On Anchieta Island, bees were collected in the first year along the Praia das Palmas trail (23° 32′ 25.0″ S and 45° 04′ 15.5″ W, at sea level) and during the second year along the Represa trail (23° 32′ 27.3″ S and 45° 03′ 58.9″ W, 18 m.a.s.l.). The distance between these trails is approximately 450 m.

In the first year, male orchid bees were attracted with cineole, eugenol, and vanillin. These compounds are considered to be the most effective for attracting males of most euglossine species (Dressier 1982a; Pearson and Dressier 1985; Rebêlo and Garófalo 1997). In order to verify the occurrence of species in which males had not been attracted by the fragrances used, in the second year, the following aromatic compounds were utilized: amyl acetate, benzyl acetate, benzyl benzoate, methyl benzoate, β-ionone, β-myrcene, ethyl butyrate, methyl cinnamate, phenethyl alcohol, linalool, and methyl salicylate. These procedures were made in both study areas.

Bees were netted when arriving at absorvent paper wads soaked with the chemical baits. The paper wads were suspended from branches by a string 1.5 m aboveground and arranged least 4.5 m from each other along the sampling trails. The paper wads were replenished every 60 minutes with 1 mL of chemical to prevent losses due to their volatility. In addition to the individuals collected when arriving at baits, females and males observed on flowers in the vicinity of the study trails, females collecting materials to build nests (such as clay and resin), and cleptoparasitic females searching for nests to attack were also captured. All individuals were killed in 96% ethanol and preserved in this solution for subsequent molecular analysis (Rocha-Filho et al. 2013). All specimens were deposited in the Collection of Solitary Wasps and Bees in the Department of Biology of the University of São Paulo, Ribeirão Preto. The identification of specimens was based on the keys published by Kimsey (1979, 1982), Dressier (1982b), BonillaGómez and Nates-Parra (1992), Rebêlo and Moure (1996), Oliveira (2006), Faria Jr and Melo (2007), and Nemésio (2009), and followed the species distribution criteria presented in Moure's Bee Catalogue (Moure et al. 2008).

### Statistical Analysis

Rarefaction curves were constructed to assess whether species richness differed between areas or between different stations in each area, and whether species dominance differed across study sites. In the rarefaction analysis, which estimates species richness for a standardized number of individuals, the total abundance of males of each species was used. Rarefaction curves were calculated using the program EcoSim (Gotelli and Entsminger 2004).

To quantify species diversity based on the number of males collected, the ShannonWiener index was used, and indices were compared using Hutcheson's *t*-test (Hutcheson 1970). Uniformity indices were calculated following Pielou (1966). The dominant species at each study site was determined by using the Simpson's index and the BergerParker dominance index. The Sørensen (Sørensen 1948) and Jaccard coefficients were used to compare community composition between the study areas. The quantitative similarity coefficient of Morisita (1959) was used to analyze the similarity in the fauna of the two areas based on the relative abundance of the males collected. All of these tests were performed using the program Bio-Dap (Magurran 1988). A cluster analysis using the UPGMA method (Unweighted Pair Group Method using Arithmetic Averages; Romesburg 1984) in the MVSP 3.1 statistical program was conducted in order to compare our results to previously published data from other areas of São Paulo state. Cluster analysis was performed using the binary Sorensen similarity matrix, which ignores abundances and thus minimizes potential biases caused by differences among sampling efforts across studies.

## Results

A total of 1,575 individuals of 23 species in four genera (*Eufriesea, Euglossa, Eulaema*, and *Exaerete*) were captured (Table 1). In Picinguaba, the values of abundance and species richness were 951 and 20, respectively. On Anchieta Island, 624 individuals of 17 species were collected (Table 1).

Of the 23 species recorded, three (*Ef. auriceps, Ef. mussitans*, and *Ef. violacea*) were found only on the island, and six (*Ef. danielis, Ef. dentilabris, Eg. iopoecila, Eg. pleosticta, Eg. townsendi*, and *Ex. smaragdina*) were observed only in Picinguaba (Table 1). Of the 11 species sampled in the second year in Picinguaba, only three (*Eg. roderici, Eg. securigera*, and *El. helvola*) were new records. Of the ten species collected during the second year on Anchieta Island, only four (*Ef. auriceps, Eg. roderici, Eg. securigera*, and *El. helvola*) were attracted exclusively during this survey period (Table 1).

The qualitative similarity coefficients, Jaccard (J = 0.61) and Sorensen (S = 0.76), both had relatively high values. In contrast, Morisita's coefficient (*C_λ_*), which considers the quantitative data, was low (*C_λ_* = 0.48), as the abundance recorded in Picinguaba (N = 951) was considerably higher than that recorded on Anchieta Island (N = 624).

The Shannon-Wiener diversity index (H) was significantly higher (t = 11.91, *p* < 0.05) for the community in Picinguaba (H′ = 2.09) than for that on Anchieta Island (H′ = 1.35).

The Berger-Parker dominance index (d) was high for the island community (d = 0.63), which was dominated by *Eg. cordata*. Dominance was lower in Picinguaba (d = 0.23), where the most abundant species, *Eg. Iopoecila*, represented only 23% of all species sampled. Likewise, the Simpson index (S) differed considerably between the sampling sites, indicating a lower diversity in the island communities (S = 0.43) than in the communities recorded in Picinguaba (S = 0.16). This pattern was also reflected in the Pielou's evenness index (J′), which demonstrated a lower evenness on Anchieta Island (J′ = 0.48) than in the more uniform Picinguaba (J′ = 0.70).

**Figure 1.**
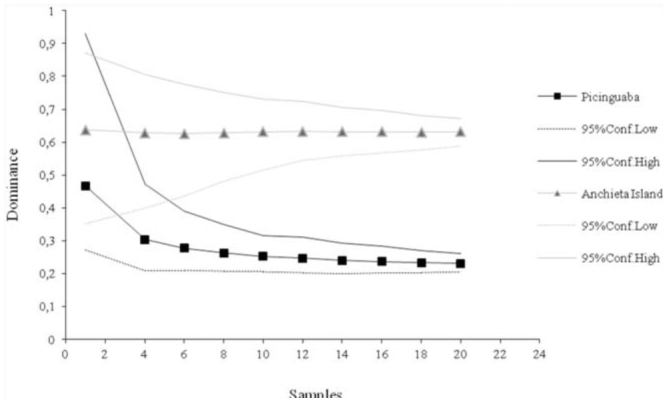
Rarefaction curves (from 1000 simulations) and associated 95% confidence intervals of species dominance in the Picinguaba and Anchieta Island euglossine communities. High quality figures are available online.

**Figure 2.**
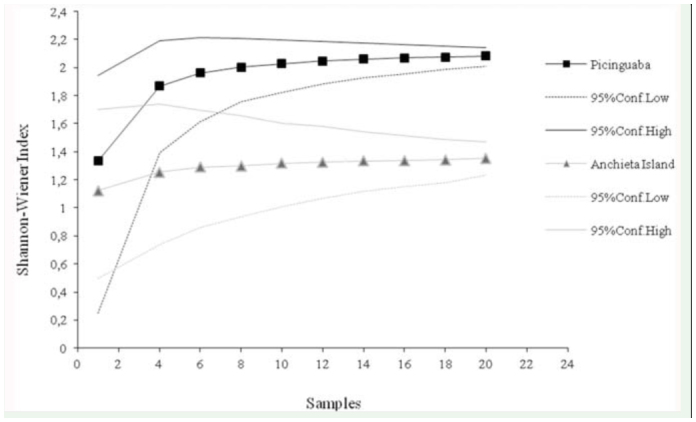
Rarefaction curves (from 1000 simulations) and associated 95% confidence intervals of diversity (the Shannon-Wiener diversity index) in the Picinguaba and Anchieta Island euglossine communities. High quality figures are available online.

**Figure 3.**
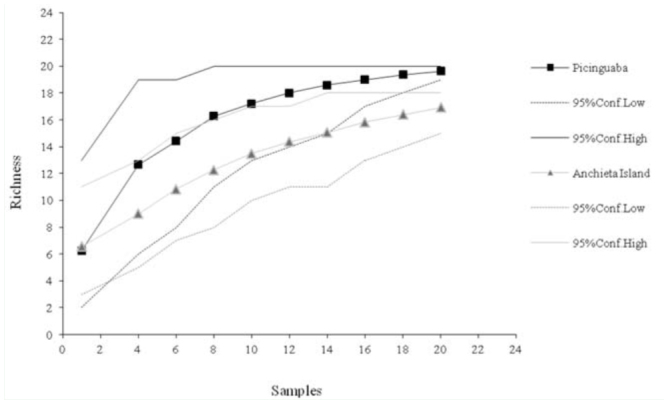
Rarefaction curves (from 1000 simulations) and associated 95% confidence intervals of species richness in the Picinguaba and Anchieta Island euglossine communities. High quality figures are available online.

The difference in dominance between the two sampled euglossine communities was apparent in the rarefaction curves (Figure 1), which indicated that the dominance values were significantly higher for Anchieta Island, beginning with the sixth collection. Similarly, the 95% confidence intervals of the rarefiedShannon-Wiener index did not overlap, beginning with the eighth collection (Figure 2), revealing a significant difference in diversity between the communities from the two study areas. However, the species richness curves did not differ significantly between study areas, as there was no separation between the 95% confidence intervals generated for each area (Figure 3).

**Figure 4.**
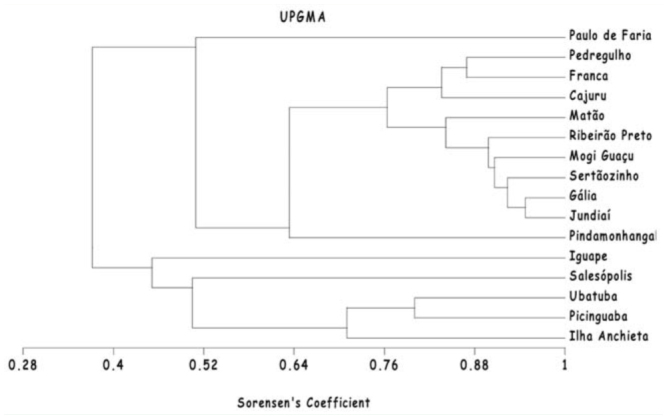
Dendrogram constructed from Sorensen coefficients calculated for the euglossine communities sampled throughout São Paulo State. “Ubatuba” represents data from the study by Singer and Sazima (2004) in the region of Picinguaba; “Picinguaba” and “Ilha Anchieta” refer to data obtained in the present study.The other localities are: Salesópolis (Wilms 1995), lguape (Knoll et al. 2004), Pindamonhangaba (Uehara-Prado and Garófalo 2006), Jundiaí (Garófalo et al. 1998), Gália (Serrano and Garófalo 2008), Sertãozinho (Rebêlo and Garófalo 1997), Mogi Guaçu (Camillo et al. 2000), Ribeirão Preto (Jesus and Garófalo 2004), Matão (Jesus and Garófalo 2000), Cajuru (Rebêlo and Garófalo 1991, 1997), Franca (Nascimento et al. 2000), Pedregulho (Mateus et al. 1993), Paulo de Faria (Braga and Garófalo 2000). High quality figures are available online.

**Figure 5.**
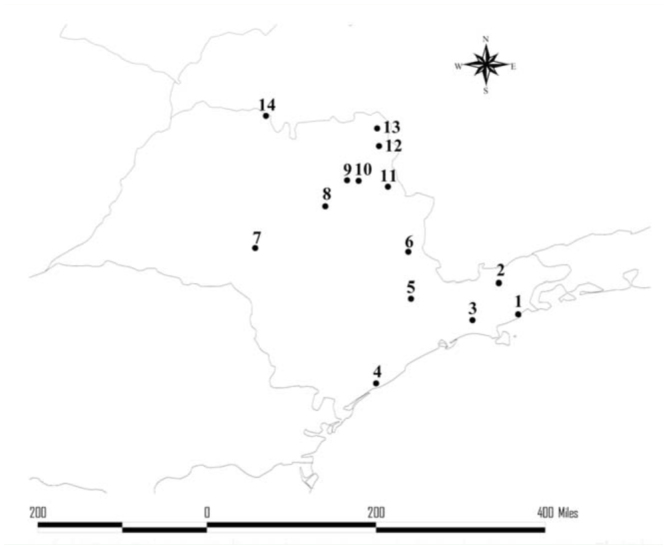
Map of euglossine study locations in São Paulo State. 1 — Ubatuba (present study and Singer and Sazima 2004); 2 — Pindamonhangaba (Uehara-Prado and Garofalo 2006); 3 — Salesópolis (Wilms 1995); 4 — lguape (Knoll et al. 2004); 5 — Jundiaí (Garófalo et al. 1998); 6 — Mogi Guaçu (Camillo et al. 2000); 7 — Gália (Serrano and Garófalo 2008); 8 — Matão (Jesus and Garófalo 2000); 9 — Sertãozinho (Rebêlo and Garófalo 1997); 10 — Ribeirão Preto (Jesus and Garófalo 2004); 11 — Cajuru (Rebêlo and Garófalo 1991, 1997); 12 — Franca (Nascimento et al. 2000); 13 — Pedregulho (Mateus et al. 1993); 14 — Paulo de Faria (Braga and Garófalo 2000). High quality figures are available online.

The UPGMA cluster analysis revealed high similarity between the two studies conducted in the region of Picinguaba (note the proximity of “Ubatuba” and “Picinguaba” in the dendrogram in Figure 4). Anchieta Island appears as a sister group to Ubatuba and Picinguaba, and these three areas form a distinct cluster together with the areas studied in Iguape and Salesópolis (the former is a coastal area, and the latter borders the coastal municipalities of Bertioga, Caraguatatuba, and São Sebastião; Figure 5). Paulo de Faria appears as a sister group to the other areas studied in the interior of São Paulo state. Some of these interior sites, such as Pedregulho and Franca, are highly similar to each other. The comparison between Gália and Jundiaí yielded the highest Sørensen coefficient value of all comparisons (Figure 4).

Four species, *Eg. cordata, Eg. iopoecila, Eg. sapphirina*, and *El. cingulata*, accounted for 83.87% of the individuals collected. Despite the similarity in community composition indicated by the Jaccard and Sorensen coefficients, the relative abundance of each species differed considerably between the two areas. Collections were dominated by *Eg. iopoecila, Eg. sapphirina, Eg. cordata*, and *El. cingulata* in Picinguaba, while *Eg. cordata, Eg. stellfeldi, El. cingulata*, and *El. seabrai* were the most abundant species collected on Anchieta Island.

## Discussion

The set of euglossine bee species recorded in the present study, particularly in Picinguaba(Table 2), was very similar to that found by Singer and Sazima (2004). Although those authors reported a total of 15 euglossine species, correcting for taxonomic misclassification and a female *Ex. smaragdina* that was collected but not included in the study, the actual total was 20 species. Except for two female *Euglossa mandibularis* (Friese) collected on flowers and male *Euglossa viridis* (Perty) captured in eugenol, all species documented by Singer and Sazima (2004) were also recorded in the present study. We also captured *Ef. auriceps, Ef. surinamensis, Eg. truncata, Eg. townsendi*, and *El. helvola*, which were not recorded by Singer and Sazima (2004).

The species richness (Table 2) was higher than what has been documented at other Atlantic Forest locations in the state of São Paulo (Mateus et al. 1993; Rebêlo and Garófalo 1991, 1997; Wilms 1995; Garófalo et al. 1998; Braga and Garófalo 2000; Camillo et al. 2000; Jesus and Garófalo 2000, 2004; Nasci-Nascimento et al. 2000; Knoll et al. 2004; Singer and Sazima 2004; Uehara-Prado and Garófalo 2006; Serrano and Garófalo 2008). This increased richness may be a consequence of our use of a greater variety of odors as scent baits, the duration of the monthly sampling (6 hrs), which was longer in the present study, and our additional records of euglossine specimens visiting flowers of plants found near the bait sites. According to Rebêlo and Garófalo (1997), obtaining data from specimens on flowers and the use of trap-nests are important methods for increasing knowledge about the euglossine fauna of a region.

The dendrogram derived using Sørensen coefficients reflects the similarity between the species found in Picinguaba in the present study and those found by Singer and Sazima (2004). Data from studies carried out in locations along or near the coast (Iguape, Ubatuba, and Salesópolis) are relatively similar and cluster separately from the data obtained in the inland areas of São Paulo. This similarity of the communities in coastal locations is due to the presence of several species, including *Ef. dentilabris, Ef. mussitans, Eg. iopoecila, Eg. roderici, Eg. stellfeldi*, and *Eg. viridis*, that were not recorded at inland locations. Throughout the areas of Atlantic Forest, these species appear to be distributed predominantly at low altitudes in coastal regions. This trend is in contrast to other species endemic to this biome, including *Ef. auriceps, Ef. violacea, Euglossa annectans* Dressier, *Eg. fimbriata*, and *Eg. truncata* (Nemésio and Silveira 2007b), which also occur in the interior of São Paulo, especially at altitudes above 1,000 m in locations such as Franca and Pedregulho (Mateus et al. 1993; Rebêlo and Garófalo 1997; Nascimento et al. 2000). Conversely, several species recorded in the interior of the state (*Euglossa despecta* Moure, *Euglossa imperialis* Cockerell, *Euglossa leucotricha* Rebêlo and Moure, and *Euglossa melanotricha* Moure) were not found in the coastal area of the present study, in Iguape, or in regions near the coast such as Salesópolis (Wilms 1995) and Pindamonhangaba (Uehara-Prado and Garófalo 2006) (see Table 2). Another distinguishing factor of the inland areas was the low diversity of the genera *Eufriesea* and *Eulaema*, which were represented only by *Ef. violacea* and *El. nigrita*, respectively.

Euglossine surveys conducted in Atlantic Forest areas of Brazilian states other than São Paulo have found lower numbers of *Eufriesea* species and, with the exception of surveys in the state of Espírito Santo and along the northeast coast, have recorded fewer species of *Eulaema* and *Exaerete* (Table 3). The total of seven *Eufriesea* species found in Ubatuba makes the present study one of the most successful in terms of the species richness of this genus, even in comparison with studies conducted in other biomes such as the Amazon (Powell and Powell 1987; Becker et al. 1991; Oliveira and Campos 1995; Nemésio and Morato 2006; Rasmussen 2009), which is known to contain a richer euglossine bee community with higher levels of endemism than the Atlantic Forest (Nemésio and Silveira 2007b). Another important factor is the record of *El. helvola*, a species that is distributed from Bolivia to Central Brazil in areas dominated by cerrado (savanna) ecosystems (Oliveira 2000; Moure 2003). However, Nemésio and Silveira (2006b) collected a male *El. helvola* in central Minas Gerais state, expanding its known geographical distribution and suggesting a possible parapatric or sympatic relationship with *El. seabrai*.

Despite the similarity in species richness at the two study areas, the prevalence of *Eg. cordata* on Anchieta Island was responsible for both the low compositional uniformity between the areas and the high values of the Simpson and Berger-Parker indices on the island. Similar results were observed by Aguiar and Gaglianone (2008), whose study areas were characterized by the dominance of *Eg. cordata* and *El. nigrita*. Previous studies (Peruquetti et al. 1999; Tonhasca et al. 2002) have suggested that *Eg. cordata* is a species that is typically found in disturbed habitats. This species has a wide distributional range, occurring in almost all Brazilian biomes and even in populated areas including urban centers (Rebêlo and Moure 1996; Wittmann et al. 1998; Lopes et al. 2007; López-Uribe and Del Lama 2007; Nemésio and Silveira 2007b; Mendes et al. 2008). Indeed, *Eg. cordata* seems to be ecologically plastic, as it is also abundant in the best-preserved areas of Atlantic Forest, as documented by Peruquetti et al. (1999), Tonhasca et al. (2002), Ramalho et al. (2009), and the present study, in which it was found that *Eg. cordata* was the third most commonly recorded species in Picinguaba.

The first record of human occupation on Anchieta Island dates from 1803, when a detachment of the Portuguese army landed in order to defend the region (Marcos Carrilho 1998). Only since the creation of the PEIA in 1977 has human activity been restricted to tourism and recreation in areas delimited by management planning (Guillaumon et al. 1989; Marcos Carrilho 1998). Human presence on the island has caused the degradation of forest areas, mainly due to harvesting and subsistence activities and the introduction of exotic plant species (Guillaumon et al. 1989). Additionally, in 1983 the São Paulo Zoo introduced 100 individuals of 15 mammal species (Bovendorp and Galetti 2007). Areas of coastal (“restinga”) forest and inland tropical ombrophilous forests have been densely occupied by these invasive mammals due to the absence of any natural predators. This occupation has resulted in the loss of ground vegetation due to trampling and the formation of permanent trails (Robim 1999; Alvarez et al. 2008).

The introduction of exotic species can be a major cause of biodiversity loss because these species can alter the structure and stability of ecological communities (Courchamp et al. 2003; Richardson and Pysek 2006). This phenomenon is especially true in the case of islands, where exotic species can proliferate in an uncontrolled manner due to the lack of predators, parasites, and other natural enemies (Emmel 1976). According to Magurran (2003), an increase in the dominance of one or more species can characterize a disturbed or altered habitat such as that found on Anchieta Island. Paralleling the results obtained for the euglossine bees in the present study, Fadini et al. (2009) reported a high preva- lence of *Turdus flavipes* (Vieillot). At a density approximately four times that observed in continental Atlantic Forest areas, this species has had deleterious effects on other bird species on the island. Bovendorp et al. (2008) observed that the density of the tegu lizard *Tupinambis merianae* (Duméril and Bibron) increased from 20 individuals/km^2^ in dense forest areas to 109 individuals/km^2^ in more open areas that had suffered anthropogenic interference.

In comparison with Anchieta Island, the community composition in Picinguaba was more even and was characterized by a relatively low prevalence of the most abundant species, *Eg. iopoecila* and *Eg. sapphirina*. On Anchieta Island, the former was absent, and the latter was represented by only a few specimens. Tonhasca et al. (2002) reported similar results, as they collected a smaller number of males of these two species in disturbed areas and forest fragments than in more wellpreserved secondary forest sites. Likewise, Nemésio and Silveira (2006a) found a larger number of *Eg. sapphirina* individuals in the forest interior and suggested that this species could be considered as a bioindicator of wellpreserved environments. Our results support this idea, as more than 95% of the *Eg. sapphirina* specimens that were collected came from Picinguaba. Another possible bioindicator species is *Eg. iopoecila*, which was recorded only in Picinguaba despite an equal sample effort in both study areas. Further support for this idea comes from the fact that Anchieta Island, at 828 hectares in area, is approximately one tenth the size of Picinguaba, which covers an area of approximately 8,000 hectares. We can therefore be confident that the surveys carried out on the smaller island area provide a more representative sample of the euglossine communities that are present.

In addition to the species mentioned above, *El. cingulata, Eg. roderici*, and *Eg. ioprosopa* were also recorded less frequently on the island than in Picinguaba. Oliveira (2000) classified *El. cingulata* as a species of wellpreserved, densely forested areas, although other studies indicate that this species is also abundant in open areas and along forest edges (Tonhasca et al. 2002; Nemésio and Silveira 2006a). There are few records of *Eg. roderici* and *Eg. ioprosopa* from Atlantic Forest areas (Wilms 1995; Knoll et al. 2004; Singer and Sazima 2004; Nemésio 2009; Ramalho et al. 2009), which indicates a need for more studies on the euglossine communities present in this biome.

Despite suggestions that *Euglossa analis* Westwood (Bonilla-Gómez 1999; Tonhasca et al. 2002; Ramalho et al. 2009), *Eg. sapphirina* (Nemésio and Silveira 2006a), and *El. cingulata* (Oliveira 2000) are possible indicators of preserved environments and that *Eg. cordata* and *El. nigrita* (Peruquetti et al. 1999; Tonhasca et al. 2002) are characteristic species of open and modified habitats, no studies have yet corroborated the reliability of euglossine bees as bioindicators. However, Silva et al. (2009) demonstrated through wing morphometry that climatic and anthropogenic factors may adversely affect the stability and development of *Eg. pleosticta*, whereas *El. nigrita* appeared to be relatively resistant to such effects. Likewise, by sampling forest fragments of different sizes, Giangarelli et al. (2009) concluded that populations of *Ef. violacea* require larger areas for survival, and that the absence of this species could reflect the degree of disturbance experienced by an area, making this species a potential bioindicator.

Due to the relative ease of sampling euglossine males that are attracted to artificial aromatic baits, this group could provide useful models for future studies of environmental quality and the preservation of natural areas and conservation units (Brown 1991). As demonstrated by studies of the negative impact of habitat fragmentation, which results in biodiversity loss (Courchamp et al. 2003) and has negative effects on the euglossine communities (Powell and Powell 1987; Becker et al. 1991; Tonhasca et al. 2003; Milet-Pinheiro and Schlindwein 2005; Brosi 2009), the potential use of these species as bioindicators is promising, especially in Atlantic Forest areas. The Atlantic Forest is one of the tropical biomes that has been the most fragmented and degraded by human intervention. These activities threaten the high species diversity and the high degree of endemism of this biome (Fearnside et al. 1996; Ranta et al. 1998; Morellato and Haddad 2000; Myers et al. 2000). Myers and Knoll (2001) point out that the decline of biodiversity causes changes in natural ecosystem services, which, in addition to affecting human livelihoods, may also disrupt evolutionary processes.

**Table 1.**
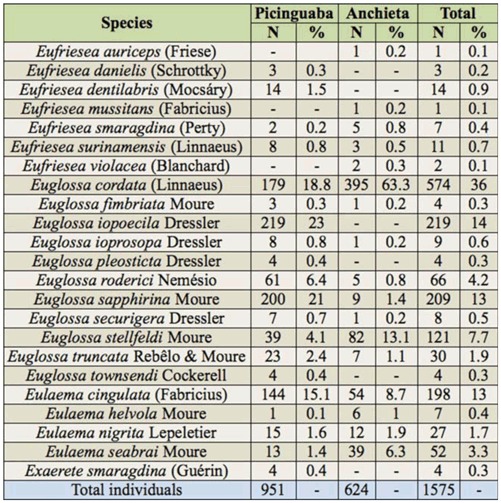
Euglossine bees collected in Picinguaba and on Anchieta Island, Ubatuba, São Paulo, from August 2007 to July 2009.

**Table 2.**
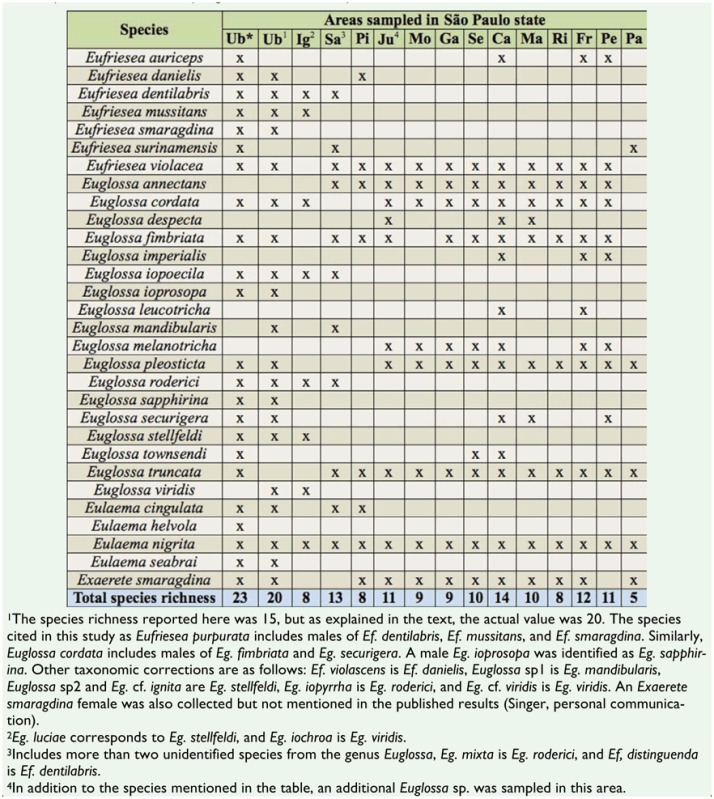
Euglossine bee species sampled in surveys conducted in the Atlantic Forest of São Paulo State. Ub* = Ubatuba (present study), Ub = Ubatuba (Singer and Sazima 2004), Ig = Iguape (Knoll et al. 2004), Sa = Salesopolis (Wilms 1995), Pi = Pindamonhangaba (Uehara-Prado and Garófalo 2006), Ju = Jundiaí (Garófalo et al., 1998), Mo = Mogi Guaçu (Camillo et al., 2000), Ga = Gália (Serrano and Garófalo 2008), Se = Sertãozinho (Rebêlo and Garófalo 1997), Ca = Cajuru (Rebêlo and Garófalo 1991, 1997), Ma = Matão (Jesus and Garófalo 2000), Ri = Ribeirão Preto (Jesus and Garófalo 2004), Fr = Franca (Nascimento et al. 2000), Pe = Pedregulho (Mateus et al. 1993), Pa = Paulo de Faria (Braga and Garófalo 2000).

**Table 3.**
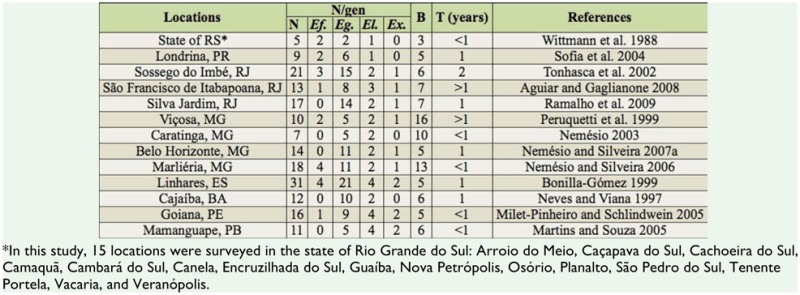
Summary of Atlantic Forest areas previously surveyed for euglossine bees in Brazil. N = Species richness, N/gen = Species per genus, B = Number of aromatic baits used, T = Duration of survey. *Ef*. = *Eufriesea, Eg*. = *Euglossa, El*. = *Eulaema, Ex. = Exaerete*.
